# The Role of Zinc Chloride in Enhancing Mechanical, Thermal and Electrical Performance of Ethylene Vinyl Acetate/Carbonized Wood Fiber Conductive Composite

**DOI:** 10.3390/polym13040600

**Published:** 2021-02-17

**Authors:** Mohd Hanif Mohd Pisal, Azlin Fazlina Osman, Tan Soo Jin, Rozyanty A. Rahman, Awad A. Alrashdi, Abdulhakim Masa

**Affiliations:** 1 Faculty of Chemical Engineering Technology, Universiti Malaysia Perlis (UniMAP), Perlis 01000, Malaysia; azlin@unimap.edu.my (A.F.O.); sjtan@unimap.edu.my (T.S.J.); rozyanty@unimap.edu.my (R.A.R.); 2 Centre of Excellence for Biomass Utilization (CoEBU), Universiti Malaysia Perlis (UniMAP), Perlis 01000, Malaysia; 3 Center of Excellence Geopolymer and Green Technology (CegeoGTech), Universiti Malaysia Perlis (UniMAP), Perlis 01000, Malaysia; 4 Chemistry Department, Al-qunfudah University College, Umm Al-Qura University, Al-qunfudah Center for Scientific Research (QCSR), Al Qunfudah 21962, Saudi Arabia; aarashdi@uqu.edu.sa; 5 Department of Rubber Technology and Polymer Science, Faculty of Science and Technology, Prince of Songkla University, Pattani 94000, Thailand; abdulhakim.m@psu.ac.th

**Keywords:** ethylene vinyl acetate, carbonized wood fiber, zinc chloride, conductive, copolymer, composite, carbon natural fiber

## Abstract

Carbonized natural filler can offer the production of low cost composites with an eco-friendliness value. The evolving field of electronics encourages the exploration of more functions and potential for carbonized natural filler, such as by modifying its surface chemistry. In this work, we have performed surface modification on carbonized wood fiber (CWF) prior to it being used as filler in the ethylene vinyl acetate (EVA) composite system. Zinc chloride (ZnCl_2_) with various contents (2 to 8 wt%) was used to surface modify the CWF and the effects of ZnCl_2_ composition on the surface morphology and chemistry of the CWF filler were investigated. Furthermore, the absorptive, mechanical, thermal, and electrical properties of the EVA composites containing CWF-ZnCl_2_ were also analyzed. SEM images indicated changes in the morphology of the CWF while FTIR analysis proved the presence of ZnCl_2_ functional groups in the CWF. EVA composites incorporating the CWF-ZnCl_2_ showed superior mechanical, thermal and electrical properties compared to the ones containing the CWF. The optimum content of ZnCl_2_ was found to be 6 wt%. Surface modification raised the electrical conductivity of the EVA/CWF composite through the development of conductive deposits in the porous structure of the CWF as a channel for ionic and electronic transfer between the CWF and EVA matrix.

## 1. Introduction

The development of high-performance polymer composites through the manufacture of glass fibers and petroleum-based materials incurs the release of a huge amount of greenhouse gases into the atmosphere [1]. To overcome this global issue, more industries are now looking for environmentally friendly materials that are recyclable, biodegradable, reusable and can be used for any purpose. The use of natural-based materials is one of the targeted strategies to achieve this goal. For instance, natural fibers are used as a reinforcement material to produce partially and fully green composites. Biobased resins and petrochemical-based resins may act as matrices in both types of composites [1,2,3].

Copolymers are those polymers that are derived from two different types of monomer. There are numerous commercially important copolymeric materials widely available including ethylene vinyl acetate (EVA). In the production of polymer composite, EVA has been utilized as a matrix material, owing to its excellent processability, high toughness, good heat retention, and flexibility. Generally, the copolymer may be produced with different concentrations of vinyl acetate (VA) content. Hence, the EVA’s processability, mechanical properties, crystallinity degree and solubility also vary with VA content [4]. It is interesting to note that impact strength, elasticity, and permeability to gases could be enhanced by increasing the VA content. In order to improve the mechanical and thermal properties of the copolymer, fillers such as nanoclay and carbon nanotubes (CNT) must be added into its structure [5]. Meanwhile, conductive particles must be added as filler in order to improve the conductivity of this copolymer as its own insulating property. Furthermore, the insulator-to-semiconductor transition can be achieved by the copolymer and it can be transformed into a so-called conductive polymer composite (CPC). CPC has attracted the attention of researchers, both from academia and industries, the world over, as it offers a wide range of applications. However, there are challenges to using CPC, which include poor oxidation and electro-active stability, along with poor mechanical properties [6].

The process of adopting synthetic fibers as a reinforcement filler may cause health problems like skin irritation and lung cancer, mainly for the manufacturers and consumers [7]. Therefore, many researchers have started to explore new alternatives for augmenting the filler with natural fibers, owing to their renewability and abundance. The application of natural fibers in the production of thermoplastic composites has offered good environmental preservation with the opportunity to improve the performance of the composites. Particularly, the benefits of using natural fibers over synthetic ones include recyclability, lower cost, biodegradability, good thermal properties, noncorrosive nature, lower specific weight, and high specific strength and stiffness [8]. Furthermore, the carbonization process can be performed by converting the natural fiber into carbon filler, where the complex substances will undergo pyrolysis into the simplest one via the heating method. Carbon filler can be used as a sustainable conductive filler in the production of the CPC. 

The dispersion states of conductive fillers in the polymeric matrix are critically important to promote good electrical conduction. Therefore, controlling the dispersion state is one of the main factors for achieving homogenous composites. The distribution of filler in the matrix can be improved by adding coupling agents, while chemical treatment can be applied to improve the capability of the filler in transmitting conductivity in the composites [9].

The addition of chemical substances like surface modifier will enhance the filler−matrix interfacial adhesion in a CPC system. According to Mohd Yazid et al. [10], the enhancement in conductivity was caused by the enhancement in filler–matrix adhesion and good distribution of polyaniline in the polyethylene oxide/polyvinyl chloride (PEO/PVC) with the presence of naphthalene as surface modifier agent. Composites with a more homogeneous morphology were obtained as a result of the surface modification process of the conductive filler.

Zinc chloride, a chemical compound abbreviated as ZnCl_2_ is an inorganic binary salt. Other names are zinc (II) chloride, zinc dichloride, or butter of zinc [11]. ZnCl_2_ has previously been used as a photo-stabilizer in the polymer composite system [12]. Furthermore, this chemical is also used for the formation of porous carbon nanofibers, beneficial in the fabrication of productive electrodes for supercapacitors [13]. In this research, we have conducted the very first attempt to use ZnCl_2_ as surface modifier of carbonized wood fiber (CWF) in order to improve its conductivity. The best loading of CWF in the EVA composite system, which is 10 phr (per hundred resin) [14] was chosen in this study. The thermal, electrical, and mechanical properties of the EVA composites containing CWF surface modified with different percentages of ZnCl_2_ were investigated and compared to the control EVA composite (with unmodified CWF).

## 2. Methodology

### 2.1. Composite Preparation and Reagents

The methods and procedures to produce the CWF filler and the EVA/CWF composites were explained in detail in our previous paper [14]. However, in this current work, the CWF was surface modified with ZnCl_2_ to allow further improvement in the electrical conductivity, mechanical and thermal properties of the EVA/CWF composite. Different ZnCl_2_ contents were employed and the best composition was determined based on the electrical conductivity, mechanical and thermal property data. In the following paragraph, we explain the chemicals and method used to prepare the surface-modified CWF.

Zinc chloride (ZnCl_2_) was used as surface modifier for the CWF and ethanol was used to dissolve ZnCl_2_. Both chemicals were supplied by AR Alatan Sdn Bhd. (Kedah, Malaysia). Firstly, 2 wt% of ZnCl_2_ in ethanol solution was prepared in a beaker. The CWF filler was then added into the solution and continuously stirred with rotor speed at 500 rpm for 24 h using magnetic stirrer model HTS-1003 supplied by Copens Scientific (M) Sdn Bhd (Selangor, Malaysia). The resultant suspension of CWF-ZnCl_2_ was dried in an oven for 24 h at 50 °C to eliminate ethanol residue. The surface modification of CWF filler was continued using various ZnCl_2_ contents: 4 wt%, 6 wt%, and 8 wt%. After that, the surface-modified CWF-ZnCl_2_ with an average diameter size of around 77 µm was used as filler in the production of EVA/CWF composite. EVA/CWF (100/10 phr) composites were prepared through melt compounding process as described earlier in our previous work [14].

### 2.2. Absorption Test

Absorption analysis was performed according to ISO 1817. Three samples were cut into 20 mm × 10 mm sheets and immersed in toluene at room temperature for 46 h. Then, the samples were taken out at fixed time intervals, dried using filter paper, and then weighed using an analytical balance of 0.1 mg resolution. The percentage of absorption was calculated using Equation (1):

(1) $$Absorption\left(\%\;\right)=\;\frac{W_{2}-W_{1}}{W_{1}}\;\times \;100\%$$

where *W*_1_ and *W*_2_ are the original dry and after exposure weights, respectively. The percentage of absorption was plotted as a function of time.

### 2.3. Scanning Electron Microscope (SEM) Analysis 

The surface morphology of the unmodified and surface-modified CWF filler, and morphology of the tensile fractured surface of the composites were analyzed using SEM model JOEL JSM-6460LA (Tokyo, Japan). Before the SEM was performed, the samples went through a sputtering coating. A thin palladium layer with a thickness of 20 µm was applied using an Auto Fine Coater to prevent electrostatic charges at the time of examination. The average particle size and particle size distribution of fillers (CWF and CWF-ZnCl_2_) was analyzed using ImageJ version 1.39 for Windows. ImageJ was developed at the National Institutes of Health (NIH), (Bethesda, MD, USA). The software is a Java-based public domain image processing and analysis program, which is freely available, open-source, multithreaded, and platform-independent [15].

### 2.4. Tensile Test

Tensile strength, modulus of elasticity, and elongation at break of all samples were evaluated using ASTM D638 tensile test, performed through Universal Testing Machine Instron 5569 (Norwood, MA, USA). The tensile test was operated at a capacity of 5 kN, gauge length of 50 mm, and crosshead speed of 50 mm/min. An average of five dumbbell shaped samples of 50 mm in length and 4 mm in width were used and acclimatized at relative humidity (30 ± 2)% and ambient temperature (25 ± 3) °C ahead of the testing.

### 2.5. Fourier Transform Infrared (FTIR) Spectroscopy Test

FTIR analysis was performed to confirm the presence of the functional group of ZnCl_2_ in the CWF. Perkin-Elmer Spectrum RX1 Series equipment (Waltham, MA, USA) was used. The powder samples were ground with 0.10 wt% potassium bromide (KBr) powder and pressed into a disc tablet. These disc tablets and selected composite samples (EVA/CWF-ZnCl_2_ with 6 wt% ZnCl_2_) were analyzed. For each sample, 20 scans were taken at a scanning range of 650–4000 cm^−1^ and resolution of 5 cm^−1^. After scanning, the FTIR curves with percentage transmittance (%T) versus wavenumber (cm^−1^) were plotted.

### 2.6. X-ray Diffraction (XRD) Characterization

The crystallinity percentage of the composite samples was obtained utilizing a Shimadzu XRD-6000 Analyzer (Kyoto, Japan) at an acceleration voltage of 35 kV and 25 mA with CuKα (λ = 1.5406 nm) radiation source. Data were recorded in the range of 5–50° (2θ) and the percentage of crystallinity values was calculated using DIFFRAC.EVA software. This analysis was operated at ambient temperature with a scan speed of 0.1 s/step for fast scanning.

### 2.7. Electrical Conductivity Test

The electrical properties of the composites were measured using a four-probe I-V measurement system. The measurement was carried out using Keithley Model 4200 Semiconductor Characterization System (Keithley Instruments, Cleveland, OH, USA) with voltage ranging from 0 to 10 V. The conductivity (σ) was calculated using Equation (2), where *L* is the distance between electrodes in cm, *A* is the cross-sectional area in cm^2^, and *R* is the electrical resistance in Ω.

(2) $$\sigma =\left(\frac{L}{AR}\right)$$

### 2.8. Thermogravimetric Analysis (TGA)

Thermal degradation analysis was performed using a PerkinElmer Pyris TGA7 (Waltham, MA, USA) applying ASTM D3850-2000. Samples with a mass of ± 10 mg were heated at 100 to 900 °C using a heating rate of 20 °C/min under flowing nitrogen of 50 mL/min. Thermal properties, namely 5% of weight loss (T_−5%wt_), 50% of weight loss (T_−50%wt_), residual mass, and max decomposition temperature (T_−Max%wt_) were determined using TGA and DTG curves.

## 3. Results and Discussions

### 3.1. Absorption Properties

The absorption properties of EVA/CWF composites comprising different compositions of ZnCl_2_ are presented in Table 1. The absorption percentage of the EVA/CWF composite in organic solvent (toluene) reduced when the ZnCl_2_ composition increased from 2 to 8 wt%. This was expected, as the presence of a greater amount of inorganic substance in the EVA/CWF structure would surely restrict the permeation of organic solvent molecules into the matrix. For instance, the absorption percentage of the EVA-CWF with 2 wt% ZnCl_2_ was 189.32% but the value reduced to 187.81% when 8 wt% ZnCl_2_ was employed. Furthermore, interphase bonding between filler and matrix affects the absorption of a composite [16]. It is believed that ZnCl_2_ played the role of surface modifier to improve the interphase bonding between the CWF and EVA matrix. This would reduce the pathways for toluene diffusion through the matrix. 

### 3.2. Morphology Analysis

**Figure 1:**
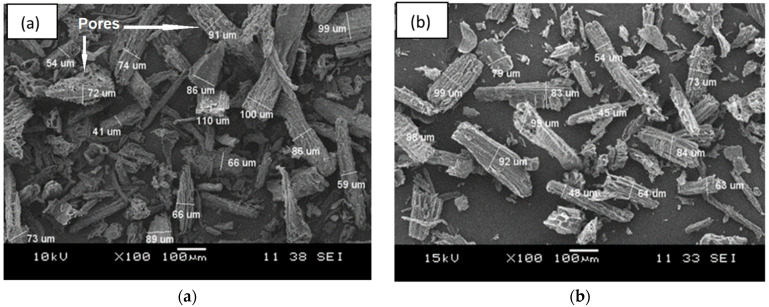
The SEM micrographs of filler (**a**) CWF and (**b**) CWF-ZnCl_2_.

**Figure 2:**
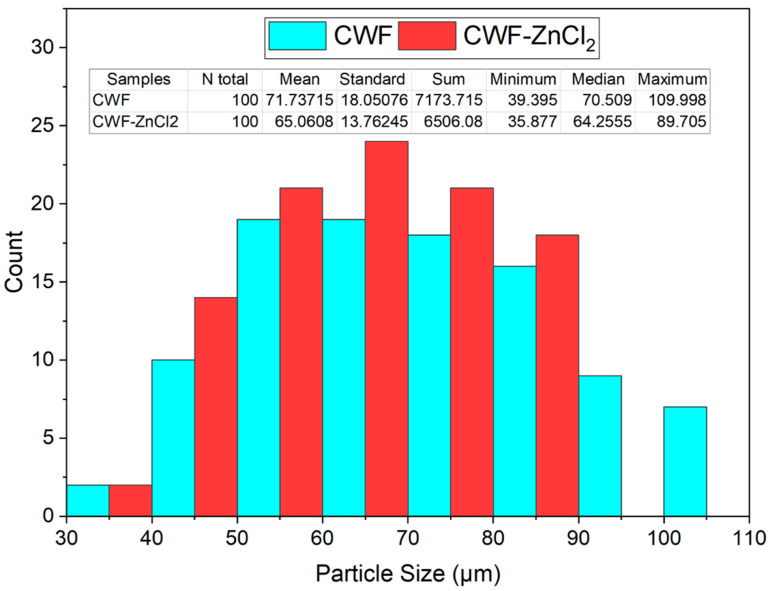
Histogram of particle size distribution of CWF and CWF-ZnCl_2_ filler.

Figure 1 shows the SEM surface morphology, while Figure 2 presents the particle size distribution histogram of the CWF and CWF-ZnCl_2_ fillers at 6 wt% of ZnCl_2_ content. Based on the SEM images, we can see roughly that the particle size of the CWF was slightly reduced after being surface modified with 6 wt% ZnCl_2_. The particle size and particle size distribution data are summarized in a histogram to allow clear comparison on particle dimensions of both samples (Figure 2). The mean particle size of the CWF and CWF-ZnCl_2_ was 72 µm and 65 µm, respectively, showing that the particle size has slightly been reduced due to a long stirring time during the preparation of the CWF-ZnCl_2_. Disaggregation of some agglomerated CWF particles could happen, therefore finer fibers could be seen in the CWF-ZnCl_2_ filler. However, disaggregation of CWF widened the particle size distribution of the CWF-ZnCl_2_ filler. This was because the level of disaggregation was also affected by other factors such as the amount of deposited carbonaceous substance on the surface of each particle of the CWF, which also affected the size of the individual particles. Both surface morphologies possessed randomly distributed grooves, small flaws, and irregular forms of the fiber surfaces. It can be said that the original textures were still maintained upon surface modification and a similar observation was reported by Singh and Choudhary [17]. However, the appearance of pores on the surface of the CWF particles was more obvious than on the surface of the CWF-ZnCl_2_ particles. Deposition of carbonaceous substances on the structure of the CWF-ZnCl_2_ could eliminate the pores. This is further discussed in the XRD results section.

**Figure 3:**
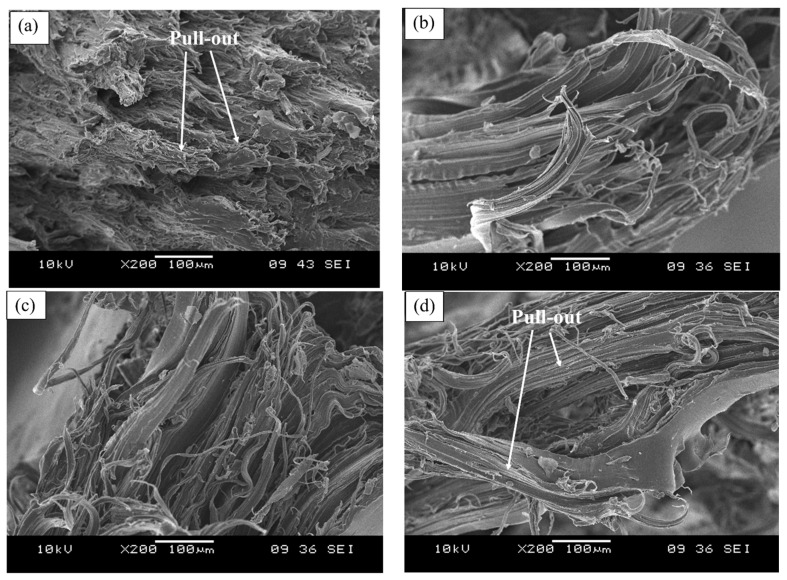
Tensile fractured surface of EVA/CWF composites containing different contents of ZnCl_2_: (**a**) 0, (**b**) 2 wt%, (**c**) 6 wt%, and (**d**) 8 wt%.

Figure 3 illustrates the micrograph of the tensile fractured surface of the EVA composites containing the unmodified CWF and CWF-ZnCl_2_ with 2, 6, and 8 wt% ZnCl_2_ content. As can be seen in Figure 3a, the tensile fractured surface exposed the pull-out of the CWF particles from the matrix. Obviously, the presence of gaps and voids resulted in the appearance of a nonhomogeneous and rough surface morphology. A similar phenomenon was observed in a composite comprising untreated wood [18]. However, upon surface modification with ZnCl_2_, the appearance of gaps and voids related to fiber pull out reduced. Another observation is related to matrix deformation (stretching) of the composites upon the application of the tensile forces. Apparently, the composites containing ZnCl_2_ showed more homogeneous matrix deformation resulting from a more homogenous mixture of the EVA copolymer and CWF-ZnCl_2_ filler. These indicated good interphase bonding between the EVA matrix and CWF-ZnCl_2_ filler. This result was in good agreement with the findings of Liu et al. [19].

### 3.3. Tensile Properties

**Figure 4:**
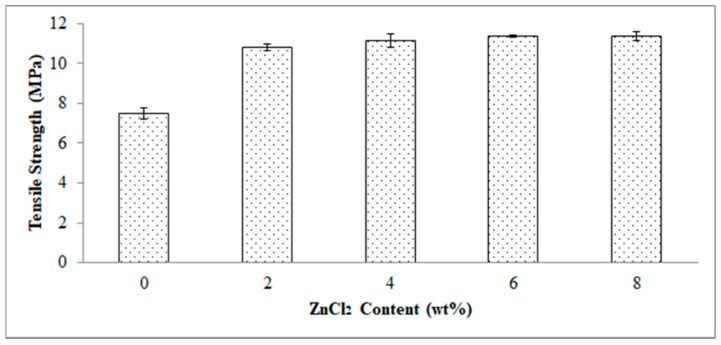
Effect of ZnCl_2_ content on tensile strength of EVA/CWF composites.

Figure 4 demonstrates the effect of ZnCl_2_ content on the tensile strength of the EVA/CWF composites. Indicatively, the tensile strength increased by 44.60, 47.66, 49.61, and 49.70% as the ZnCl_2_ content employed was 2, 4, 6 and 8 wt%, respectively. Based on these values, it can be concluded that the optimum content of ZnCl_2_ to surface modify the CWF filler in the EVA/CWF composite is 6 wt%. This is because as the ZnCl_2_ content increased to 8 wt%, an intangible increment was seen. Filler and matrix had distinctive microstructures and physical properties. Surface modification of CWF could enhance the interfacial bonding between both materials to a certain extent. This allowed a mechanical interlocking mechanism through the permeation of the EVA molecules into the narrow cavities of the CWF-ZnCl_2_ filler surface during the flow of the EVA melting in the compounding process [20]. Better interface interaction between EVA copolymer and CWF-ZnCl_2_ particles helped to dissipate stress from the matrix to the filler, which contributed to the higher tensile strength of the composites. On the other hand, this chemical alteration affected the chemical bonding between matrix and filler as well as their dispersion in the EVA matrix during the compounding phase. The tensile strength reached an optimum increment when the 6 wt% of ZnCl_2_ content was used to surface modify the CWF. Surface modification promoted good association between the EVA matrix and CWF-ZnCl_2_ filler, resulting in improved interfacial interactions between both constituents. Furthermore, the CWF surface modified with ZnCl_2_ was more mechanically stable due to reduced porosity as a result of graphene layer deposition (see XRD result). Therefore, it provided greater reinforcement to the EVA matrix.

**Figure 5:**
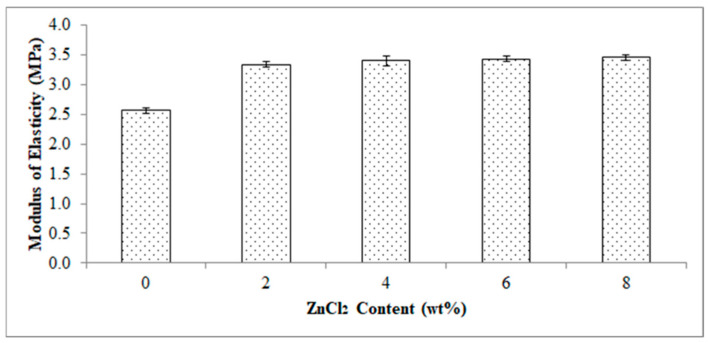
Effect of ZnCl_2_ content on modulus of elasticity of EVA/CWF composites.

**Figure 6:**
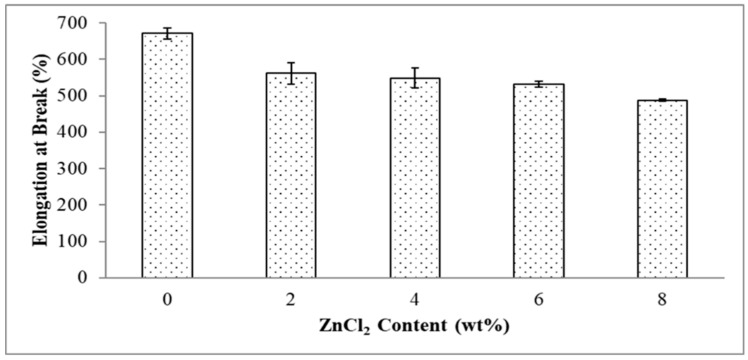
Effect of ZnCl_2_ content on elongation at break of EVA/CWF composites.

Figure 5 reports the effect of ZnCl_2_ content on a modulus of elasticity of the EVA/CWF composites. The composite showed an increase in modulus of elasticity value by 31.58, 33.58, 34.46 and 35.04% when ZnCl_2_ was added at 2, 4, 6 and 8 wt%, respectively. The increment in modulus of elasticity was owing to the inclusion of more rigid CWF-ZnCl_2_ particles in the matrix and thus resulted in stiffer composites. Tharayil et al., [21] reported a similar observation in which the modulus of elasticity of low-density polyethylene (LLDPE) was improved with the incorporation of 7 to 20 wt% zinc oxide (ZnO) powder. This was a result of the stiffening effect induced by the ZnCl_2_ modified CWF filler, as the metal chloride enhanced the interaction between the EVA phase and CWF filler thus resulting in a higher stiffness than the pure composite. Moreover, the movement of the polymer chains was deprived by the inclusion of CWF-ZnCl_2_ resulting from the enhancement in the rigidity of the composites. Deformability of the rigid interphase between matrix and filler was attributed to the decline in elongation at break as shown in Figure 6.

Figure 6 demonstrates the effect of ZnCl_2_ content on elongation at break of the EVA/CWF composites. The stiffening effect caused by the surface modification of the CWF also aligned with the elongation at break values of the composites. The composite showed a decreasing trend when the ZnCl_2_ content used to surface modify the CWF increased from 2 to 8 wt%. Elongation at break value reduced by 19.58, 21.91, 24.96 and 34.06% when the ZnCl_2_ content was 2, 4, 6 and 8 wt%, respectively. It can be concluded that the incorporation of the ZnCl_2_ enhanced the compatibility between the EVA/CWF phases by the formation of the chemical bridge between the matrix and filler. This led to greater stiffness and lower chain mobility, hence lower elongation at break values when more content of ZnCl_2_ was added. The homogenous dispersion of CWF-ZnCl_2_ in the matrix and strong interaction between both constituents had resulted in the flow restriction of the EVA matrix when the polymer molecules passed one another. Thus, higher, more rigid composites were obtained. The similar phenomenon was observed by Rusu et al. [22], in which well-interacted high-density polyethylene (HDPE) and zinc filler significantly enhanced the rigidity of the matrix.

### 3.4. Fourier Transform Infrared (FTIR) Analysis

**Figure 7:**
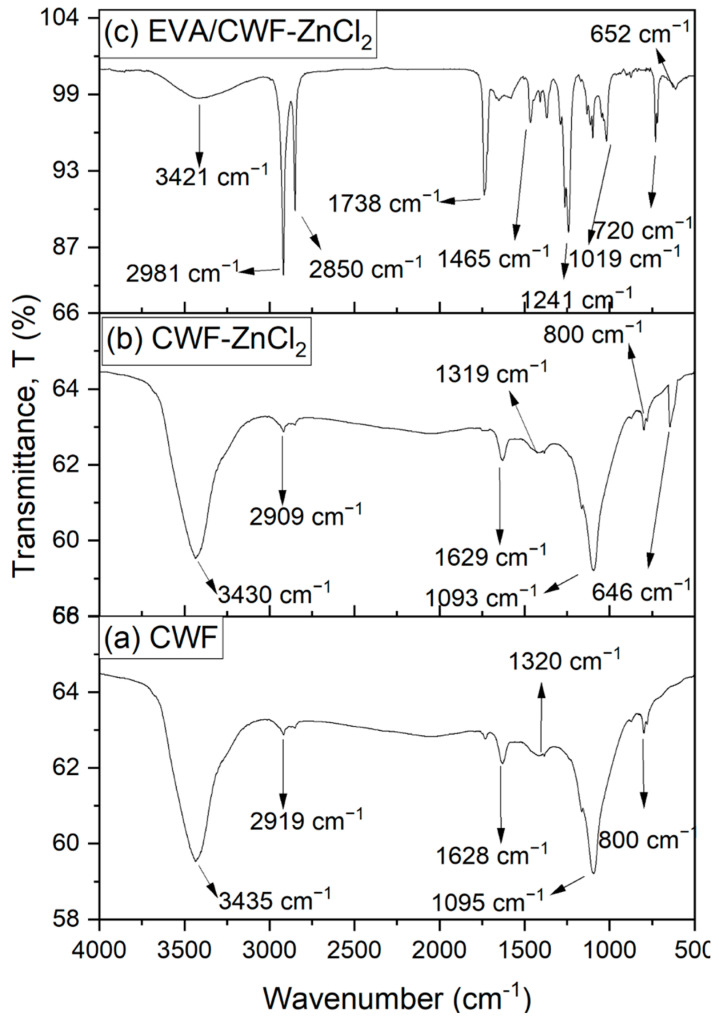
Infrared spectroscopy spectrum of (**a**) CWF filler, (**b**) CWF-ZnCl_2_ filler and (**c**) EVA/CWF-ZnCl_2_ composites at 6 wt% of ZnCl_2_ content.

Figure 7b presents the FTIR spectrum of the ZnCl_2_ modified CWF filler at 6 wt% content. When benchmarked with the CWF filler, the spectra of the CWF modified with ZnCl_2_ were shifted downward at wavenumbers: 3430, 2909, 1629, 1319, 1093, and 800 cm^−1^. These characteristic peaks of cellulose corresponding to a strong presence of O–H stretching, C-H stretching vibration, C=O stretching of the amide group, C–H asymmetric deformation in methyl, and phenolic alcohol, C–O deformation in secondary alcohol of the aliphatic ether, and C–H bending. Similar peaks were observed by Trivedi et al. [23]. However, the appearance of a peak at wavenumber 646 cm^−1^ in the CWF-ZnCl_2_ filler indicates the stretching of metal-halogen Zn-Cl due the attachment of the surface modifier on the surface of the CWF. This peak, however, was absent in the unmodified CWF filler (Figure 7a). This proved the success of surface modification in the CWF-ZnCl_2_ filler. It has successfully formed a bridge between the CWF filler and the ZnCl_2_.

**Figure 8:**
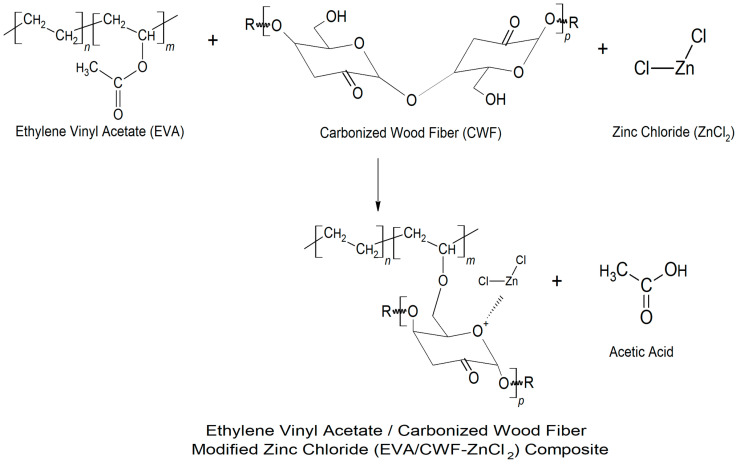
Illustration of the mechanism of interaction between the CWF-modified ZnCl_2_ (CWF- ZnCl_2_) filler with the EVA matrix of the EVA/CWF-ZnCl_2_ composite.

Figure 7c demonstrates the FTIR spectrum of the EVA/CWF composites at 6 wt% content of ZnCl_2_. Due to the surface modification of CWF filler with ZnCl_2_, the absorption spectra of the filler showed a new peak at 3421 cm^−1^, indicating the presence of the OH groups. Mostly, ZnCl_2_ is very sensitive to water, because ZnCl_2_ itself is hygroscopic and can be considered deliquescent. Furthermore, the absorption peaks at the asymmetric and symmetric stretching intensities of 2981 and 2850 cm^−1^ were associated with alky groups from the EVA matrix. The absorption peak at 1738 cm^−1^ was attributed to C=O stretching. A peak at 1465 cm^−1^ was assigned to methylene scissoring peaks of the EVA matrix. Absorption peaks at 1241, 1019, and 720 cm^−1^ were as a result of the asymmetric and symmetric C–O–C stretching of ester groups in the VA regions. In addition, absorption peaks at 652 cm^−1^ showed metal-halogen of Zn-Cl stretching in the composites. The proposed mechanism of reaction between the EVA matrix, CWF filler, and ZnCl_2_ materials is illustrated in Figure 8. Generally, the reaction between EVA and CWF-ZnCl_2_ forms acetic acid. However, during residence time inside the Brabender mixer (under oxygen-rich environment), another reaction could be expected. As high temperature was used for the melt compounding process, acetic acid further reacts with hydroxyl (OH) groups of the CWF to form a thermally unstable ester. In the melt state of the EVA, this acid also could react with the reactive metal sites of the composite (Zn–Cl–O), forming zinc oxide compound. The unstable ester can easily decompose when exposed to high temperature and lead to the formation of pores and nonflammable gas that subsequently cause the foaming and deposition of carbonaceous compound. The porous structure of CWF can be a good template for the deposition of carbon-zinc oxide compound, and at the same time allows the inclusion of the EVA molecular chains. Therefore, it can be a channel for ionic and electronic transfer between the CWF and EVA matrix. Good interphase bonding between CWF and EVA will promote electron mobility along the copolymer chains. These are the reasons behind the mechanical, thermal, and electrical properties enhancement observed in the EVA/CWF-ZnCl_2_ composite, especially the ones with high ZnCl_2_ content (6 and 8 wt%).

### 3.5. X-ray Diffraction (XRD) Analysis

**Figure 9:**
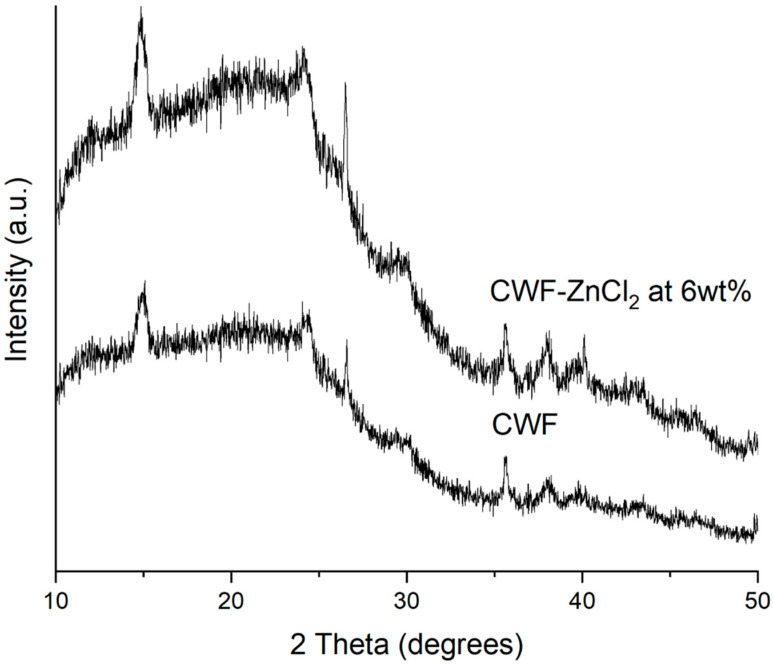
XRD diffractogram of CWF and CWF-ZnCl_2_ at 6 wt% ZnCl_2_ content.

The XRD patterns of the CWF and CWF-ZnCl_2_ filler containing 6% ZnCl_2_ are shown in Figure 9. The XRD signal of the carbonized wood fiber exhibited the typical pattern for cellulose-I structure, in which two clear peaks at 2θ = 15° and 2θ = 25° appear and can be assigned to a crystallographic plane of cellulose. This means that the pyrolysis done on this lignocellulosic material (wood fiber) for carbonization did not lead to complete destruction of the cellulose crystallite, but only resulted in degradation of the cellulose into smaller fragmented structures while retaining its parent crystallite form. Furthermore, the XRD pattern also indicated the presence of two broad and diffuse bands centered at ~2θ = ~22 and ~43°, associated with principal graphite diffraction (from 002 and 100/101 set of planes, respectively) [23]. These bands also corresponded to the interlayer spacing and microcrystallite lateral dimension of the turbostratic (fully disordered) graphene layers. However, instead of the above described broad and diffused bands, several well-developed peaks could be observed at 2θ = ~26, 29, 36, 38 and 40°.

These peaks were more intense in the XRD signal of the CWF-ZnCl_2_. This shows that the graphene layers exist in the form of stacks [24] and much higher in the case of the CWF surface modified with ZnCl_2_ (CWF-ZnCl_2_). This could be related to the decarboxylation process that takes place in the CWF due to the reaction between metal salt (ZnCl_2_) and ethanol. Decarboxylation resulted in the formation of the active graphene that would be grafted onto the cellulose structure of the CWF. Consequently, the deposition of the graphene on the surface of the CWF occurred. As seen in the SEM image (Figure 1), the CWF-ZnCl_2_ possessed a less porous structure than the CW, due to the deposition of more graphene layers on the surface of the CWF-ZnCl_2._ Due to this factor, the CWF-ZnCl_2_ became more mechanically stable and conductive, thus it is a better filler than the unmodified CWF.

**Figure 10:**
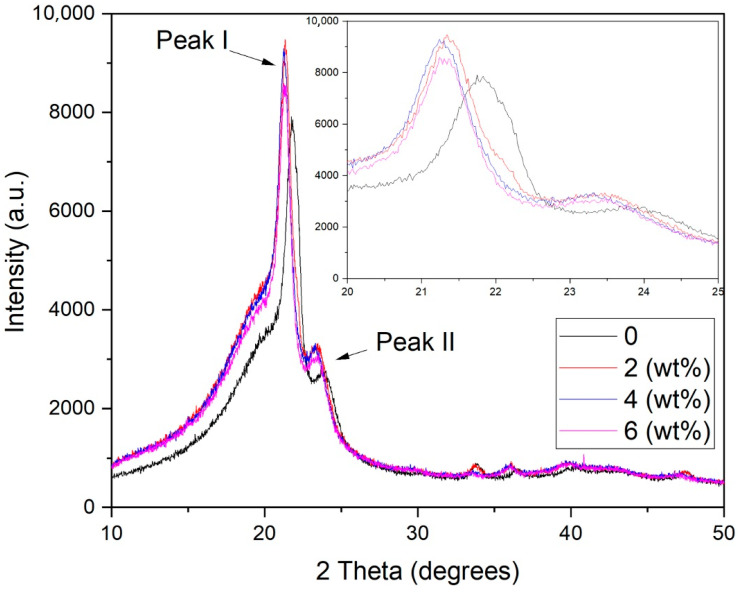
XRD diffractogram of EVA/CWF composites at different ZnCl_2_ contents.

As revealed in Figure 10, the XRD diffractogram of the EVA/CWF composites at different ZnCl_2_ contents showed a single crystalline and broad peak at 2θ angles of 21.50 (±0.2) (Peak I) and 23.60 (±0.3) (Peak II) due to the crystalline structure of the EVA matrix. Based on the results, the addition of ZnCl_2_ into the CWF filler caused a slight reduction in the intensity of Peak I. This was in agreement with the obtained percentage of crystallinity values (see Table 2), in which the addition of ZnCl_2_ led to the reduction of the crystallinity of the EVA/CWF composite. A similar observation was made by Trivedi et al. [23]. However, a more intense Peak II can be seen in the EVA/CWF-ZnCl_2_ as opposed to the EVA/CWF composite. As previously shown in Figure 9, the CWF-ZnCl_2_ filler exhibited a more intense peak in the region of 2θ = 22–25° than the CWF filler, due to the deposition of the graphene on its surface. When incorporated into the EVA matrix, the XRD signal of the resultant EVA/CWF-ZnCl_2_ composite showed the superimposed peaks of the polyethylene crystalline structure of the EVA and the CWF-ZnCl_2_ filler, affecting the region of 2θ = 22–25°, where Peak II is located. As a result, a more intense Peak II can be observed. However, a reduction in the intensity of both Peak I and Peak II could be seen when the ZnCl_2_ content of the CWF-ZnCl_2_ filler increased from 2 to 8 wt%. The lowest percentage of crystallinity was observed in the EVA/CWF-ZnCl_2_ composite with 8% ZnCl_2_ content. The reason behind this was that the presence of a greater amount of ZnCl_2_ resulted in more disruption in the crystalline order of the ethylene phase of the EVA matrix. This was because more chemical bridges would be developed between the CWF and EVA matrix by the metal salt, leading to reduced mobility of the ethylene molecular chains of the EVA matrix. Consequently, prohibitive rearrangement of the ethylene chains occurred, slowing down the formation of crystallites. Eventually, a lower percentage of crystallinity could be seen in the composites with higher content of ZnCl_2_. However, with lower degree of crystalline structure, the electron transferring capability of the matrix was better because there were more amorphous regions for more mobile transfer of electrons. The results of the electrical conductivity tests presented in the next section have proved this. 

### 3.6. Electrical Conductivity

**Figure 11:**
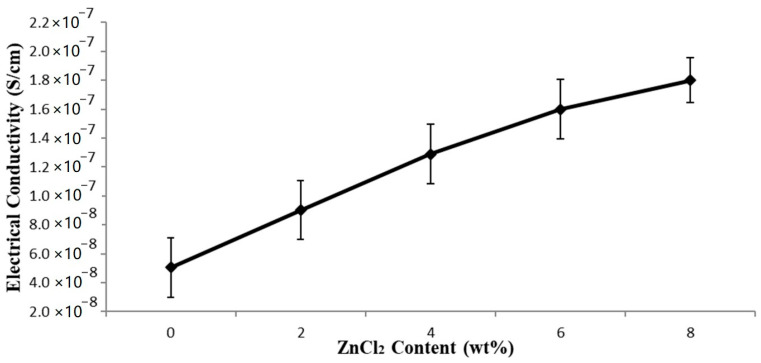
Effect of ZnCl_2_ content on electrical conductivity of EVA/CWF composites.

The electrical conductivity of the EVA/CWF composites (with and without ZnCl_2_) is shown in Figure 11. Generally, there is an interesting fact that can be highlighted from this data. The EVA/CWF composite containing CWF-ZnCl_2_ possessed much higher electrical conductivity values compared to the one containing the unmodified CWF. The electrical conductivity values of the composite improved further with the inclusion of a higher content of ZnCl_2_. The increments of 78.26, 121.28, 145.31, and 157.81% were observed when the ZnCl_2_ levels employed were 2, 4, 6 and 8 wt%, respectively. As mentioned previously, surface modification using ZnCl_2_ improved the capability of the CWF filler in transferring electrons due to the alteration of the porous structure of the CWF into a better channel for ionic and electronic transfer between the CWF and EVA matrix. The deposition of a graphene layer on the surface of the CWF-ZnCl_2_ also improved the capability of this filler in transferring electrons. 

Furthermore, improved interactions between the EVA matrix and CWF-ZnCl_2_ filler also allowed good transfer of electrons, as more conductive pathways were created in the whole structure of the composites that permitted the electrons to move. Another possibility could be due to size reduction of the CWF particles upon surface modification by ZnCl_2_. Particle size reduction increases the surface area of the CWF-ZnCl_2_ filler in the EVA matrix, thus providing greater conductive sites and capacity for electron transfer. Zhang et al. [25] revealed that the increment of conductivity of a polyvinyl alcohol (PVA) polymer was gained by supplementing a small value of sodium chloride that would create an increment in net charge density, leading to a decrement in the fiber diameters and increased electrical conductivity of the polymer. A similar observation was made by Kim et al. [13] where the nanofiber diameter decreased with the rise in ZnCl_2_ content resulted in the increment of the conductivity of the polyacrylonitrile (PAN) polymer solution.

### 3.7. Thermal Degradation

**Figure 12:**
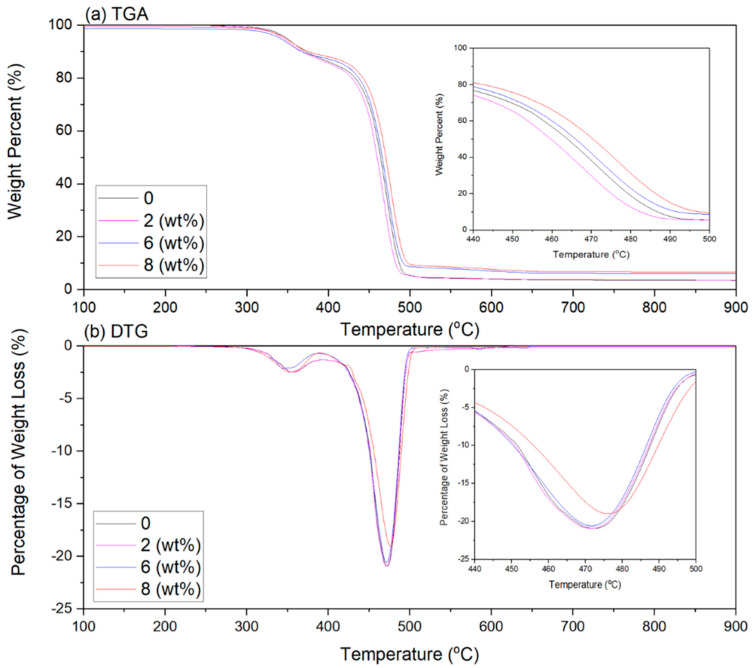
(**a**) TGA and (**b**) DTG thermogravimetric of EVA/CWF composites at different ZnCl_2_ contents.

The differential thermogravimetric (DTG) and thermogram (TGA) graphs of the EVA/CWF-ZnCl_2_ composites are presented in Figure 12, while Table 3 shows the temperature at 5% weight loss (T_−5%wt_), the temperature at 50% weight loss (T_−50%wt_), final decomposition temperature (T_−Max%wt_) and residual mass for all the composites. Generally, the EVA/CWF composites (with and without ZnCl_2_) showed a weight loss over the two-stage decomposition process, which were: (i) de-acetylation of the EVA copolymer to acetic acid and polyacetylene which occurred at a temperature between 315 °C to 390 °C and (ii) the main decomposition process which occurred at temperatures of 410 °C to 500 °C, due to decomposition of ethylene chains.

The initial degradation temperature T_−5%wt_ of the composite reduced around 0.56% due to the presence of CWF-ZnCl_2_ in its structure. However, the T_−50%wt_, T_−Max%wt_ and residual mass increased around 0.30, 0.18 and 5.80% when compared to the composite with the CWF filler. As discussed earlier, the particle size of the CWF reduced after surface modification. The CWF-ZnCl_2_ filler with smaller size and higher surface area particles might form greater interfacial bonding with the EVA matrix [26,27]. Therefore, a more thermally stable composite was produced. A similar observation was obtained by Kim et al. [13] when they incorporated 1, 3, and 5 wt% of ZnCl_2_ into polyacrylonitrile (PAN) polymer solution. The thermal stability of the PAN improved due to the presence of the inorganic salt that enhanced the physical bonding and promoted the interaction between filler and matrix, hence creating an obstacle that decelerated the volatilization of the composites over the heating process. Another related finding was observed by Rusu et al. [22], which indicated that the vicat softening temperature rose with the increase of Zn powder content in high density polyethylene (HDPE) composites. 

Other than thermal stability, the inclusion of ZnCl_2_ also increased the residual mass of the EVA/CWF composite. This could be related to the increase in the density of the filler in the composite due to the reduction in its particle size and increment in the surface area. A comparable phenomenon was studied by Mani et al. [28]. They found that as the particle size of the wheat straw fiber decreased, the char yield of the sample increased due to the increase in volatile matter and fixed carbon content. Another reason could be due to the inclusion of the ZnCl_2_ surface modifier that induced the aromatization of carbon, eliminated water, and acted as a dehydrating agent [29].

The intensity of the DTG peak (Figure 12b) relates to the rate of mass loss of the tested samples [5]. It depends on the amount of degradation product released, which is attributed to the degree of interaction between the EVA matrix and the filler (CWF vs. CWF-ZnCl_2_). Apparently, when the EVA was incorporated with surface-modified CWF (CWF-ZnCl_2_), a lower rate of mass loss during the second step of mass loss (Peak II) could be observed, especially in the ones containing 6 and 8 wt% of ZnCl_2_. This might be due to the longer time needed to break the backbone of the copolymer, as the mobility of molecular chains of the copolymer was more restricted when interacting with the CWF-ZnCl_2_. This phenomenon was due to stronger interfacial interactions developed between the matrix and filler when the CWF-ZnCl_2_ was employed as filler.

## 4. Conclusions

In this article, we highlight the great potential of natural fibers from renewable resources to produce conductive polymer composites (CPC) for possible use in electronic packaging applications. The effects of using ZnCl_2_ as a surface modifier of the CWF on the properties of the resultant EVA/CWF composite were investigated. The inclusion of CWF surface modified with ZnCl_2_ (CWF-ZnCl_2_) resulted in a reduced absorption capacity of the composite upon immersion in organic solvent (toluene). Tensile strength, modulus of elasticity, thermal stability, and electrical conductivity of the EVA/CWF composite containing CWF-ZnCl_2_ were greater than the EVA composite containing the unmodified CWF. The optimum composition of ZnCl_2_ was found to be 6 wt%. SEM analysis indicated less pores were present on the surface of the CWF-ZnCl_2_ compared to the CWF. More homogeneous matrix deformation could be seen in the composite with CWF-ZnCl_2_. XRD analysis indicated a stronger signal of graphene composition in the CWF-ZnCl_2_, which could be due to the deposition of graphene on the surface of the CWF-ZnCl_2_. The alteration of the porous structure of the CWF upon surface modification could be the reason for these property enhancements. During the melt compounding process, high temperature and oxidative conditions could result in the formation of carbon-zinc oxide compound deposition in the porous structure of the CWF-ZnCl_2_. At the same time, the EVA copolymer chains could also enter the pores and interact with the filler. Good electron transfer between the matrix and filler occurred.

## Figures and Tables

**Table 1:** Percentage absorption of EVA/CWF composites at different ZnCl_2_ contents.

ZnCl_2_ Content (wt%)	Absorption (%)
0	191.81 ± 1.27
2	189.32 ± 0.93
4	188.30 ± 1.03
6	188.02 ± 1.04
8	187.81 ± 1.27

**Table 2:** Percentage crystallinity of EVA/CWF composites at different ZnCl_2_ content.

ZnCl_2_ Content (wt%)	Crystallinity (%)
0	42.7
2	41.9
6	40.1
8	39.1

**Table 3:** Data of T_-5%wt_, T_-50%wt_, T_-Max%wt_ and residual mass of EVA/CWF composites at different ZnCl_2_ content.

ZnCl_2_ Content (wt%)	T_−5%wt_ (°C)	T_−50%wt_ (°C)	T_−Max%wt_ (°C)	Residual Mass (%)
0	348.12	469.40	475.59	4.55
2	346.18	467.99	474.75	4.83
6	346.81	468.51	475.13	5.15
8	347.14	471.23	476.61	5.34

## Data Availability

The data presented in this study are available on request from the corresponding author.
